# Global Prevalence of COVID-19-Associated Mucormycosis (CAM): Living Systematic Review and Meta-Analysis

**DOI:** 10.3390/jof7110985

**Published:** 2021-11-18

**Authors:** Salman Hussain, Abanoub Riad, Ambrish Singh, Jitka Klugarová, Benny Antony, Hasanul Banna, Miloslav Klugar

**Affiliations:** 1 Czech National Centre for Evidence-Based Healthcare and Knowledge Translation (Cochrane Czech Republic, Czech EBHC: JBI Centre of Excellence, Masaryk University GRADE Centre), Institute of Biostatistics and Analyses, Faculty of Medicine, Masaryk University, Kamenice 5, 625 00 Brno, Czech Republic; abanoub.riad@med.muni.cz (A.R.); klugarova@med.muni.cz (J.K.); klugar@med.muni.cz (M.K.); 2 Department of Public Health, Faculty of Medicine, Masaryk University, Kamenice 5, 625 00 Brno, Czech Republic; 3 Menzies Institute for Medical Research, University of Tasmania, 17 Liverpool St, Hobart, TAS 7000, Australia; ambrish.singh@utas.edu.au (A.S.); benny.eathakkattuantony@utas.edu.au (B.A.); 4 International Clinical Research Center, Molecular Control of Cell Signaling Group, St. Anne’s University Hospital, 656 91 Brno, Czech Republic; hasanul.banna@fnusa.cz

**Keywords:** coinfection, COVID-19, epidemiology, meta-analysis, mucormycosis, mycoses, prevalence, risk factors, systematic review

## Abstract

Mucormycosis, a secondary fungal infection, gained much attention in the ongoing COVID-19 pandemic. This deadly infection has a high all-cause mortality rate and imposes a significant economic, epidemiological, and humanistic burden on the patients and healthcare system. Evidence from the published epidemiological studies showed the varying prevalence of COVID-19-associated mucormycosis (CAM). This study aims to compute the pooled prevalence of CAM and other associated clinical outcomes. MEDLINE, Embase, Cochrane COVID-19 Study Register, and WHO COVID-19 databases were scanned to retrieve the relevant articles until August 2021. All studies reporting the prevalence of mucormycosis among COVID-19 patients were eligible for inclusion. Two investigators independently screened the articles against the selection criteria, extracted the data, and performed the quality assessment using the JBI tool. The pooled prevalence of CAM was the primary outcome, and the pooled prevalence of diabetes, steroid exposure, and the mortality rate were the secondary outcomes of interest. Comprehensive Meta-Analysis software version 2 was used for performing the meta-analysis. This meta-analysis comprised six studies with a pooled sample size of 52,916 COVID-19 patients with a mean age of 62.12 ± 9.69 years. The mean duration of mucormycosis onset was 14.59 ± 6.88 days after the COVID-19 diagnosis. The pooled prevalence of CAM (seven cases per 1000 patients) was 50 times higher than the highest recorded background of mucormycosis (0.14 cases per 1000 patients). A high mortality rate was found among CAM patients with a pooled prevalence rate of 29.6% (95% CI: 17.2–45.9%). Optimal glycemic control and the judicious use of steroids should be the approach for tackling rising CAM cases.

## 1. Introduction

Mucormycosis, as an angio-invasive infection, is caused by ubiquitous environmental fungal species of the Mucorales order, e.g., *Rhizopus arrhizus*, *Rhizomucor pusillus*, *Lichtheimia corymbifera* and *Apophysomyces variabili* [1]. The clinical presentation of mucormycosis varies according to the anatomical site of involvement including rhino-orbital-cerebral, pulmonary, cutaneous, gastrointestinal and disseminated forms [2]. It is widely depicted as a disease of the immunocompromised cohorts because its risk factors include uncontrolled diabetes mellitus, end-stage renal disease, hematologic malignancies, and solid organ transplantation [2]. Remarkably, the anatomical sites are associated with the predisposing medical conditions; for example, diabetes mellitus was found to be highly correlated with the rhino-cerebral form, whereas hematologic malignancies and organ transplants were associated with the pulmonary form [2,3].

The background incidence rates of mucormycosis have been rising globally owing to several demographic, epidemiologic, and iatrogenic risk factors over the last few decades [4,5]. However, the exact prevalence/incidence estimates of mucormycosis are unknown, and they are almost impossible to be calculable due to the fact that a tiny fraction of the cases is properly diagnosed and documented. A number of meta-analyses and large cohort studies have been aggregately published since 2005 with the aim of providing an in-depth understanding of the epidemiology of this devastating condition epidemiology [1,5]. Roden et al. 2005 conducted the first ever systematic review for mucormycosis cases that included 929 patients documented by 459 case reports/series, which were published between 1940 and 2003 [5]. Since this inaugural analysis, all the succeeding systematic reviews published in the last five years confirmed two main epidemiologic characteristics of mucormycosis: its rapidly growing incidence and its strong affinity to diabetes mellitus [6,7,8,9,10,11].

The clinical-level evidence exhibited a sharp rise in the incidence density of mucormycosis infections in a large Belgian hospital from 0.019 cases per 10,000 patient-days in 2000 to 0.148 cases per 10,000 patient-days in 2009 [12]. Similarly, Lewis et al. 2013 found that the prevalence of mucormycosis in a large American cancer centre increased from 0.06 cases per 100 autopsies in 1989 to 0.20 cases per 100 autopsies in 2008 [13]. This growing trend had been simultaneously reported in other high-income countries; Guinea et al. 2017 found that the period prevalence of mucormycosis-related hospitalisations increased in Spain from 1.2 cases per 100,000 admissions (1988–2006) to 3.3 per 100,000 admissions (2007–2015) [14]. In Switzerland, Ambrosioni et al. 2010 found that the point prevalence of mucormycosis rose from 0.57 cases per 100,000 admission-year before 2003, to 6.3 cases per 100,000 admission-year after 2003 [15]. In France, the population-level data revealed a significant increase in the cumulative incidence of mucormycosis from 0.7 cases per million persons in 1997 to 1.2 cases per million persons in 2006, yielding an annual increase of +7.4% (*p* < 0.001) [16]. The less developed economies were not supposed to suffer less from this phenomenal increase; for instance, three consecutive studies in India demonstrated that the annual incidence of mucormycosis was 12.9 cases per year in the first decade (1990–1999), 35.6 cases per year in the following five years (2000–2004), and 50 cases per year in the eighteen months of the study (July 2006–December 2007) [17,18,19]. Overall, India is proposed by Chander et al. 2018 to have 80 times the universal average of mucormycosis prevalence, with an estimate of 0.14 cases per 1000 diabetic persons [20]. This large discrepancy between the Indian and the universal averages is further supported by the estimates of the Leading International Fungal Education (LIFE) organisation which stated that the prevalence of mucormycosis worldwide ranged between 0.6 and 3 cases per million people, whereas the Indian rate was 140 cases per million people [21].

Nevertheless, developing a clear and robust case definition is an integral part of successful outbreak investigations, and disease surveillance and the lack of a non-invasive diagnostic techniques that can rapidly confirm the infection of mucormycosis precludes the exact estimation of its burden [22,23]. Kontoyiannis et al. 2016 provided a perfect example of how the case definition of mucormycosis can manipulate these estimations [24]. The authors aimed to study the period prevalence of mucormycosis-related hospitalisations through analysing the discharge reports of 560 hospitals in the United States (U.S.) between January 2005 and June 2014; they found that the prevalence of mucormycosis was 0.12 per 10,000 discharges, if the definition of mucormycosis was restricted to the need for amphotericin B or Posaconazole, and it would have been 0.16 per 10,000 discharges if the definition of mucormycosis was relaxed a bit [24]. In addition to the conventional microbiological and histopathological techniques that involve tissue biopsies, there is emerging, however primitive, evidence on the potential use of blood and serum to confirm the diagnosis of mucormycosis through PCR-based techniques [23].

Being a rare infectious disease, mucormycosis had never been nationally notifiable nor reportable in the vast majority of the countries in the world until the second wave of the coronavirus disease (COVID-19) pandemic that began in winter 2020 in the northern hemisphere [25]. In mid-May 2021, multiple Indian states declared that mucormycosis became a notifiable disease under the Epidemic Diseases Act, 1897 due to the expeditious increase in the cases that were primarily associated with COVID-19 infection [26,27,28].

Throughout the last two years, COVID-19 has been consistently reported in conjunction with a wide array of confusing extrapulmonary symptoms and complications, including neurologic symptoms (e.g., dysgeusia, anosmia, agitation, Guillain–Barré syndrome, and other neuropathies) [29,30], vascular symptoms (e.g., multisystem inflammatory syndrome, arrhythmias, myocardial injuries, and cardiogenic shocks) [31,32], gastrointestinal symptoms (e.g., diarrhoea, nausea, and abdominal discomfort) [33,34], skin-related symptoms (e.g., chilblains, viral exanthema, erythematous rashes, urticaria, acral ischemia, erythema multiforme, and purpura) [35,36], and oral symptoms (e.g., oral ulcers, cheilitis, mucositis, candidiasis, and halitosis) [37,38,39,40]. The syndromic landscape of COVID-19 is overburdened by the medical comorbidities that increase the risk of mortality among the infected patients and the odds of acquiring coinfections and super-infections [41,42,43,44]. The COVID-19 patients, especially the severely affected ones, had been frequently reported to suffer from opportunistic fungal infections, e.g., aspergillosis, candidiasis, mucormycosis, coccidioidomycosis, histoplasmosis, and blastomycosis, which might be initially confusing for the intensivists due to their clinical similarity with the typical respiratory symptoms of COVID-19 [37,43,44,45,46,47,48,49]. Therefore, antibiotic stewardship, high alertness of the medical staff and laboratory testing are imperative in the critical care of COVID-19 patients [45,48].

On 1 December 2020, the first report of the U.S. Centers for Disease Control and Prevention on COVID-19-associated fungal infections was released, highlighting COVID-19-associated pulmonary aspergillosis and the sporadic increase in *Candida auris* and invasive candidiasis during the ongoing pandemic [50]. In the following months, this list was expanded to include other infections such as the syndemic of COVID-19-associated mucormycosis (CAM) that was first reported in India, where this term had been coined and the largest number of cohorts and individual cases were reported so far [45,47]. The worldwide mucormycosis incidence among COVID-19 patients (active or recovered cases) is rising [51,52], and the evidence provided by the recent epidemiological studies exhibits varying incidence rates of CAM [53,54,55,56].

Heretofore, a sizeable number of studies had convincingly suggested CAM as a potential threat for health systems worldwide amid this pandemic and that it may contribute to excess mortality rates, especially in low-income settings [8,47,57]. In response to the uncertainties around this overwhelming syndemic, this living systematic review and meta-analysis was designed to compute the pooled global prevalence of CAM, which may serve as aiding evidence for health systems while they navigate through this pandemic.

## 2. Materials and Methods

This meta-analysis was conducted by adhering to the updated Preferred Reporting Items for Systematic Reviews and Meta-Analyses (PRISMA) and Meta-analysis of Observational Studies in Epidemiology (MOOSE) reporting guidelines [58,59] (Supplementary File S1).

### 2.1. Eligibility Criteria

We included cross-sectional or longitudinal studies reporting the prevalence of mucormycosis among confirmed COVID-19 patients (based on RT–PCR reports). Only studies with confirmed mucormycosis cases diagnosed based on the Centers for Disease Control and Prevention criteria, i.e., either through the histopathology, culture, or staining techniques among COVID-19 patients, were qualified for inclusion. Case reports, case series, reviews or studies with incomplete information about the confirmed number of mucormycosis cases from the total number of COVID-19 patients were excluded.

### 2.2. Information Sources

The literature search conducted in August 2021 aimed to find both published and unpublished studies. An initial limited search was conducted in MEDLINE (Ovid) and Embase (Ovid), using keywords and index terms related to COVID-19 and mucormycosis. Following an analysis of the text words contained in the title and abstract and the index terms used to describe the articles, we performed a second search across all included databases. The bibliography of relevant reviews was hand searched for any additional articles, followed by citation tracking of all the relevant articles.

The literature search was not restricted to any language. We extensively searched the following databases: MEDLINE (Ovid), Embase (Ovid), Cochrane COVID-19 Study Register, and the WHO COVID-19 database until 16 August 2021.

### 2.3. Search Strategy

The search strategy comprised medical subject heading terms related to coronavirus such as coronavirus* OR corona virus* OR 2019-ncov OR ncov19 OR ncov-19 OR 2019-novel CoV OR SARS-CoV2 OR SARS-CoV-2 OR sarscov2 OR sarscov-2 OR SARS-2-nCoV OR SARS-2-Cov OR SARS-CoV-19 OR Sars-coronavirus2 OR Sars-coronavirus-2 OR SARS 2 coronavirus* OR Severe Acute Respiratory Syndrome-CoV-2 OR SARS-like coronavirus* OR coronavirus-19 OR covid19 OR COVID-19 OR COVID 2019, and mucormycosis such as mucormycosis OR mucormycoses OR mucormycose OR mucoromycosis OR mucoromycoses OR zygomycosis OR zygomycoses OR “black fungus” OR “black fungi” OR Mucorales OR mucoralean OR Absidia OR Cunninghamella OR Mortierella OR Mucor OR Apophysomyces OR Saksenaea OR Rhizopus OR Rhizomucor OR Lichtheimia OR Cokeromyces OR Actinomucor OR Syncephalastrum (Supplementary File S2).

### 2.4. Study Selection

Two reviewers (S.H. and A.R.) independently scanned all the retrieved articles initially based on title and abstract, followed by full-text readings in the later phase. All the articles were judged against the inclusion and exclusion parameters described in the eligibility criteria. The reviewers tried to resolve any confusion in the study selection process through discussion; if consensus was not reached by discussion, then the decision was made by consulting a third reviewer (M.K.).

### 2.5. Data Collection

Two independent reviewers (S.H. and H.B.) extracted the following information from all the eligible studies: study author, year, country, study period or duration, the number of COVID-19 cases, confirmation of COVID-19, the diagnosis of mucormycosis, the number of mucormycosis cases among COVID-19 patients, the presence of comorbidities, the mean age of mucormycosis patients, female (%), clinical symptoms, treatment modalities, and mortality. Any confusion in the data collection process was resolved by discussion with a third reviewer (M.K.).

### 2.6. Primary and Secondary Outcomes

The primary outcome of this meta-analysis was to compute the pooled global prevalence of CAM. The secondary outcome was to compute the pooled prevalence of diabetes, steroid use, and mortality among CAM patients.

### 2.7. Risk of Bias Assessment

The JBI critical appraisal tool for studies reporting prevalence data was used to assess the quality assessment of the included studies. This tool comprised nine questions dealing with the target population, sample size, condition measured, and statistical analysis with yes, no, unclear, and not applicable responses.

### 2.8. Living Review

A living systematic review is a form of evidence synthesis which is updated regularly when sufficient new evidence becomes available [60]. Two reviewers will independently search the updated published and unpublished evidence every six months and include all relevant articles as per the previously described inclusion criteria. The meta-analysis will be updated continuously depending on the availability of new evidence and will be published as a separate publication.

### 2.9. Statistical Analysis

All statistical analyses were performed using Comprehensive Meta-Analysis software (CMA) version 2 (Biostat Inc., Englewood, NJ, USA). The pooled prevalence of mucormycosis among COVID-19 patients was computed. Heterogeneity was identified if the I^2^ statistics value was ≥50% or *p* < 0.10 based on the *x*^2^ test for Cochran’s Q statistics [61]. In the presence of significant heterogeneity, a meta-analysis was performed using a random effect model.

Funnel plots and meta-regressions were not performed due to the limited number of included studies. A sensitivity analysis was performed using the leave-one-out method to assess if pooled effect estimates were influenced by any single study alone. The subgroup analysis was performed based on diabetes as a comorbidity, and steroid exposure. The certainty of the assessment of CAM prevalence was assessed using GRADE (Grading of Recommendations Assessment, Development and Evaluation) approach [62]. GRADE rates the body of evidence based on the risk of bias, imprecision, inconsistency, indirectness, and publication bias.

## 3. Results

### 3.1. Study Selection

**Figure 1:**
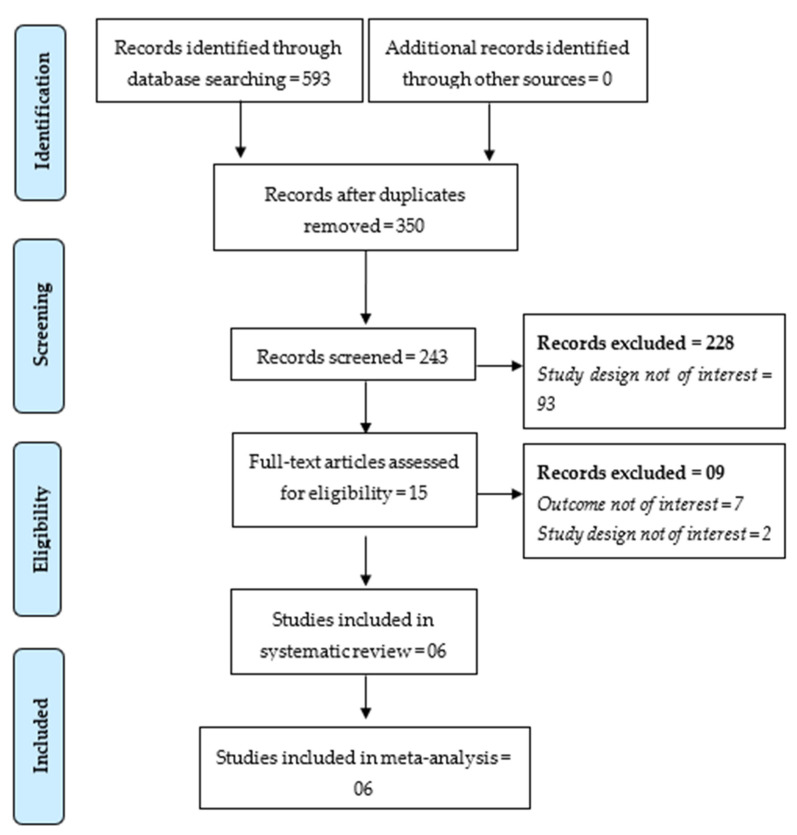
PRISMA Flowchart Displaying the Study Selection Process.

The database search gave 593 hits, of which 243 articles were retrieved for initial screening after removing 350 duplicate articles. A total of 15 full-text articles qualified for inclusion, and only six studies met the inclusion criteria for inclusion in the meta-analysis. A list of articles excluded in the full-text screening phase is presented in Supplementary File S3. A PRISMA flowchart showed the study inclusion process in the meta-analysis (Figure 1).

### 3.2. Study Characteristics

Only six studies reported the prevalence of CAM (223 patients) among a pooled sample size of 52,916 COVID-19 patients with a mean age of 62.12 ± 9.69 years [53,54,55,56]. All the included studies were designed as cross-sectional studies. COVID-19 was confirmed based on reverse transcription polymerase chain reactions (RT–PCR) in all the included studies except the study by Ramaswami et al. 2021, and mucormycosis was confirmed based on histopathology, culture or staining [53]. The majority of the studies reporting CAM prevalence were from India (*n* = 4) [53,54,55,56], whereas a single study was from Turkey [63] and Pakistan [64]. The sample size of COVID-19 patients varied noticeably, ranging from 953 to 32,814. The mean duration of mucormycosis onset was 14.59 ± 6.88 days after the COVID-19 diagnosis (Table 1).

### 3.3. Risk of Bias in Studies

All the included studies were of high quality as per the assessment based on the JBI critical appraisal tool for prevalence studies. All included studies were based on an adequate sample size with a detailed description of patients and settings. An appropriate method was used for the identification of patients and statistical analysis (Table 2).

### 3.4. Global CAM Prevalence

**Figure 2:**
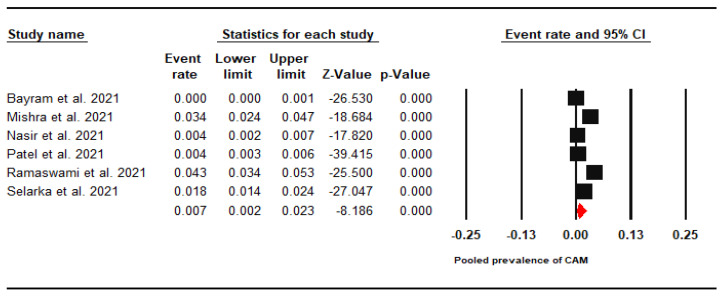
Forest Plot of the Pooled Global Prevalence of CAM.

The included studies reported prevalence in the range of 0.03–4.25%. The pooled prevalence of CAM was found to be 0.70% (95% CI: 0.2–2.3%). Due to significantly high heterogeneity (I^2^ = 98.6%), a meta-analysis was performed using a random effect analysis (Figure 2).

Headache and ophthalmological complications were the most common clinical symptoms, and pansinusitis was the most common form of paranasal sinus involvement. Liposomal amphotericin B (LAmB) was given to almost every patient, followed by surgical debridement in the majority of CAM patients.

### 3.5. Diabetes Prevalence

**Figure 3:**
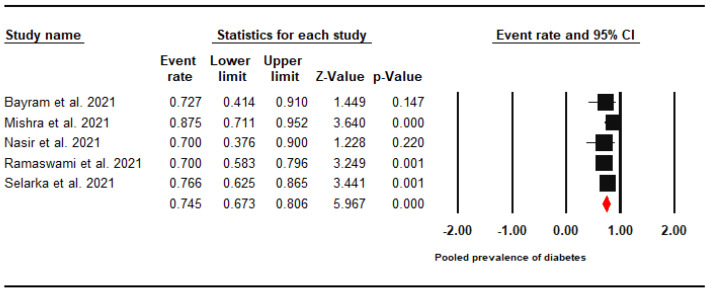
Forest Plot of the Pooled Prevalence of Diabetes Mellitus among CAM Patients.

A subgroup analysis revealed a high prevalence of diabetes with a pooled prevalence of 74.5% (95% CI: 67.3–80.6%). A meta-analysis was performed using a fixed effects model (I^2^ = 0%) (Figure 3).

### 3.6. Steroid Use

**Figure 4:**
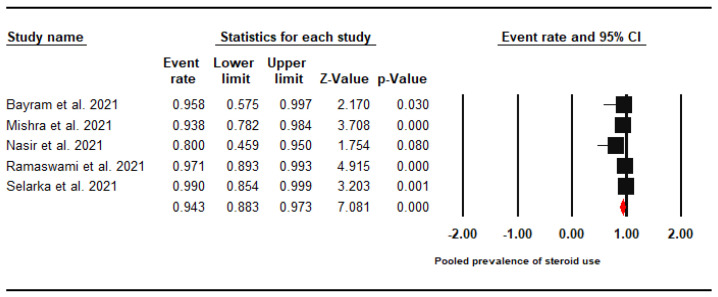
Forest Plot of the Pooled Prevalence of Steroids Use among CAM Patients.

Likewise, the pooled prevalence of steroid exposure among CAM patients was found to be 94.3% (95% CI: 88.3–97.3%, I^2^ = 31.2%) (Figure 4).

### 3.7. All-Cause Mortality Rate

**Figure 5:**
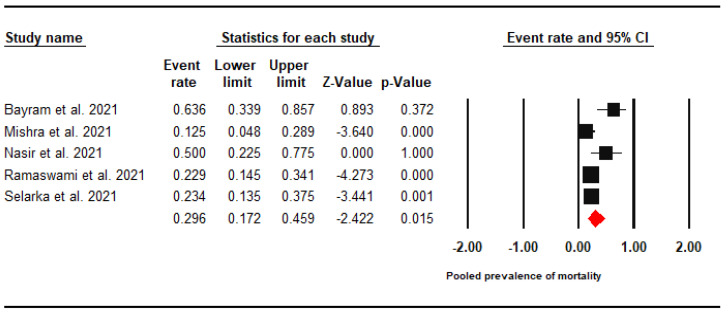
Forest Plot of the Pooled Prevalence of Steroids Use among CAM Patients.

High mortality was found among CAM patients with a pooled prevalence rate of 29.6% (95% CI: 17.2–45.9%, I^2^ = 68.3%) (Figure 5).

### 3.8. Certainty of Evidence

According to the GRADE rating system, the strength of evidence on the pooled prevalence of CAM was found to be of low certainty because of inconsistency (the presence of high heterogeneity) and no feasibility in assessing publication bias due to the limited number of included studies.

## 4. Discussion

The present meta-analysis revealed that the pooled prevalence of CAM among hospitalised COVID-19 patients was 7 cases per 1000 patients. In their comprehensive review of fungal coinfections among COVID-19 cohorts, Peng et al. (2021) found that the pooled prevalence of all fungal coinfections was 12 cases per 1000 patients, with a statistically significant difference between Asian (15 cases per 1000 patients) and European populations (7 cases per 1000 patients) [65]. The pooled prevalence of COVID-19-associated pulmonary aspergillosis (CAPA) was 6 cases per 1000 patients, which was slightly similar to what we have found in terms of CAM prevalence, and the difference between Asian (13 cases per 1000 patients) and European populations (0.1 cases per 1000 patients) was statistically significant [65].

In comparison with the background prevalence of mucormycosis in comorbid populations, the pooled prevalence of CAM was 50 times higher than the highest documented prevalence of mucormycosis that was among the diabetic patients in India (0.14 cases per 1000 patients) [20,66].

The pooled prevalence of diabetes mellitus among CAM patients was 74.5% which is similar to what had been previously reported in Mexico and Iran, where diabetes mellitus was a notable predisposing risk factor among mucormycosis patients with an overall prevalence of 72% and 75.4%, respectively [67,68]. The role of diabetes mellitus as a risk factor was less expressed in European countries, e.g., Italy (18%), France (23%), and Greece (29%), thus suggesting the role of other comorbidities in this region, especially hematologic malignancies [69,70,71]. Diabetes was also highly prevalent among CAPA patients (80%) [72]. On the other side, hematologic malignancies were reported in only two CAM patients (0.89%), yielding a much lower prevalence level than what was reported in Mexico (13.6%), India (6.3%), and Iran (3.4%) among mucormycosis patients [4,67,68].

In total, 94.3% of CAM patients received steroid therapy, thus indicating the clinical severity of their COVID-19 infection. In a recent Cochrane review, Wagner et al. 2021 concluded with moderate certainty that systemic corticosteroids could probably reduce all-cause mortality in symptomatic COVID-19 patients [73]. As of 4 August 2021, the National Institutes of Health (NIH) recommend the use of corticosteroids to control the systemic inflammatory responses that lead to lung injury and multiorgan dysfunction in severely ill COVID-19 patients, and this recommendation was packed with a meta-analysis suggesting that corticosteroids can reduce all-cause mortality and the duration of mechanical ventilation [74,75]. Steroids can be blamed in part for the mucormycosis infection through their immunomodulating role in COVID-19 patients, especially those with diabetes mellitus who are susceptible to peripheral microthrombi [76]. In oncologic patients, a cumulative prednisone dose of >600 mg is sufficient for initiating mucormycosis infection, and recipients of solid organ transplants need a dose as high as 2–7 mg of methylprednisone to get mucormycosis [77,78]. Likewise, a recent systematic review revealed that 53% of CAPA patients were on steroid therapy; therefore, a hypothetical correlation was suggested to exist between steroid use and the development of CAPA infection [79,80]. Ritter et al. 2021 challenged this hypothesis through their single-centre experience, as they found that the hazard ratio of secondary infections due to corticosteroid administration was only 1.45 (CI 95%: 0.75–2.82; *p* = 0.28) among critically ill COVID-19 patients; therefore, they recommended that corticosteroids as an effective therapy for COVID-19 should not be discontinued due to the suspicions of secondary infection [81].

The all-cause mortality rate among CAM patients was 29.6%, which is lower than the reported mortality rate of mucormycosis in the U.S. (50%), India (45%), Iran (40.8%), and South Korea (33%) [19,82,83,84]. According to Roden et al. 2005, the mortality rate of mucormycosis is highly dependent on the site of involvement (clinical type), where the disseminated type was the most fatal (96%), followed by the gastrointestinal (85%), and the pulmonary (76%) forms which were not commonly present among CAM patients [5]. Another explanation for the reduced mortality of CAM patients is attributed to the definition of mucormycosis infection among COVID-19 cohorts that is supposed to be “proven” according to the revised guidelines of the European Organisation for Research and Treatment of Cancer (EORTC) [85]. Proven fungal infections are those that occur in either immunocompromised or immunocompetent patients and are confirmed by means of histopathology or microbiology [85,86]. According to a recent report from the biennial meeting of EORTC and ECMM, the current epidemiologic evidence confirms that narrowing the definition of invasive fungal infections (IFI) from proven/probable/possible IFI to proven/probable IFI yields a sharp decline in the mortality estimates [87].

### 4.1. Strengths and Limitations

The strength of this meta-analysis was an exhaustive literature search and inclusion of only confirmed CAM patients, which makes the evidence more robust. All the studies from India had a unique set of patients as the study period and the hospital centres varied. The potential limitation could be the low generalisability of the findings, as the data was mainly from Indian settings. However, CAM cases were reported mainly in India.

### 4.2. Implications

The findings of this meta-analysis warrant future epidemiologic studies on CAM patients to precisely report the vaccination rates and characteristics among all COVID-19 patients under investigation in order to facilitate the evaluation of the role of vaccines in modifying the risks of morbidity and mortality.

## 5. Conclusions

This meta-analysis provides the first comprehensive evidence on the reported prevalence of CAM among COVID-19 hospitalised cohorts. The pooled prevalence of CAM (7 cases per 1000 patients) was comparable to the pooled prevalence of CAPA (6 cases per 1000 patients) and 50 times higher than that of the highest recorded background of mucormycosis (0.14 cases per 1000 patients). Diabetes mellitus was the most common medical comorbidity (74.5%) among CAM patients, which is consistent with the pre-existing evidence from Asian countries on the common predisposing risk factors of mucormycosis. The overall mortality rate (29.6%) among CAM patients was much lower than what is known about mortality rates of mucormycosis patients, and this finding can be attributed to the site of involvement (clinical type) or the case definition (diagnosis method). We recommend that future epidemiologic studies investigate the impact of COVID-19 vaccination on the mortality rate among CAM patients.

## Figures and Tables

**Table 1:** Included Studies’ Characterstics. Global Prevalence of COVID-19-associated Mucormycosis (CAM), as of August 2021.

Study Author; Year	Country	Study Period	COVID-19 (*n*)	Mucormycosis (*n*)	Comorbidities	Confirmation of COVID-19	Diagnosis of Mucormycosis			Mucormycosis Diagnosis After COVID-19 (days)	Mean Age (years)	Female (%)	Clinical Symptoms of Mucormycosis	Treatment	Mortality
							Stain	Culture	Histopathological						
Ramaswami et al. 2021 [53]	India	6 May 2021 to 1 June 2021	1647	70	DM: 70% (*n*= 49) HTN: 24.3% (*n* = 17) CAD: 5.7% (*n* = 4) Organ transplant: 2.9% (*n* = 2) CKD: 8.6% (*n* = 6)	Rapid antigen or nucleic acid amplification test	Yes	Yes	NR	20 days (Range 13.5–25)	Median age: 44.5 years (38–55.5)	40%	Eye pain (81.4%), Swollen eyes (80%), Nasal stuffiness (38.65), Facial pain (34.3%)	Liposomal amphotericin B (97.1%), Posoconazole (2.9%)	23%
Mishra et al. 2021 [54]	India	12 April to 31 May 2021	953	32	DM: 87.5%	RT–PCR	Yes	Yes	Yes	17.28 ± 11.76 days	58.28 ± 8.57	46.9%	Headache (93.8%), Rhinorrhoea & nasal stuffiness (62.5%), Redness or eye pain (56.2%)	Liposomal amphotericin B (100%) and endoscopic debridement (93.3%)	12.5%
Patel et al. 2021 [55]	India	1 September to 31 December 2020	12,096	53	DM	RT–PCR	Yes	Yes	Yes	NR	NR	NR	NR	Liposomal amphotericin B or amphotericin B deoxycholate, surgical debridement	NR
Selarka et al. 2021 [56]	India	3 January to 27 March 2021	2567	47	DM: 76.6%; HTN: 57.4%	RT–PCR	Yes	Yes	Yes	12.1 ± 4.6 days	55 ± 12.8	25.5%	Headache (74.5%), other symptoms include diplopia (19.1%), visual disturbances (25.5.%), and ophthalmoplegia (19.1%)	Liposomal amphotericin B (100%) and endoscopic debridement (40.4%)	23.4%
Bayram et al. 2021 [63]	Turkey	March to December 2020	32,814	11	DM: 72.7% HTN: 63.63% Chronic renal failure: 27.27%	RT–PCR	Yes	Yes	Yes	14.4 ± 4.3 days	73.1 ± 7.7	18.2%	Proptosis (100%), ophthalmoplegia (63.6%), orbital pain (81.8%), conjunctival hyperemia or chemosis (81.8%), ptosis (63.6%), fixed and dilated pupil (63.6%), vision loss (63.6%), endophthalmitis (54.5%), and decreased vision (27.3%)	Amphotericin B (100%) and radical debridement (100%)	63.63%
Nasir et al. 2021 [64]	Pakistan	July 2020 to May 2021	2839	10	DM: 70% HM: 20%	RT–PCR	Yes	Yes	Yes	16 days (Range 12–20)	Median age: 63 years (Range: 33–86)	40%	NR	Amphotericin B (100%), and surgical debridement (60%)	50%

CAD: Coronary Artery Disease; CKD: Chronic Kidney Disease; COVID-19: Coronavirus Disease; DM: Diabetes Mellitus; HM: Hematological Malignancies; HTN: Hypertension; NR: Not Reported; RT–PCR: Reverse Transcriptase Polymerase Chain Reaction.

**Table 2:** Risk of Bias Assessment Using the JBI tool for Cross-sectional Studies.

Study Author, Year	Was the Sample Frame Appropriate to Address the Target Population?	Were Study Participants Sampled in an Appropriate Way?	Was the Sample Size Adequate?	Were the Study Subjects and the Setting Described in Detail?	Was the Data Analysis Conducted with Sufficient Coverage of the Identified Sample?	Were Valid Methods Used for the Identification of the Condition?	Was the Condition Measured in a Standard, Reliable Way for All Participants?	Was There Appropriate Statistical Analysis?	Was the Response Rate Adequate, and If Not, Was the Low Response Rate Managed Appropriately?
Bayram et al. 2021 [63]	✓	✓	✓	✓	✓	✓	✓	✓	✓
Mishra et al. 2021 [54]	✓	✓	✓	✓	✓	✓	✓	✓	✓
Nasir et al. 2021 [64]	✓	✓	✓	✓	✓	✓	✓	✓	✓
Patel et al. 2021 [55]	✓	✓	✓	✓	✓	✓	✓	✓	✓
Ramaswami et al. 2021 [53]	✓	✓	✓	✓	✓	✓	✓	✓	✓
Selarka et al. 2021 [56]	✓	✓	✓	✓	✓	✓	✓	✓	✓

Yes: ✓ No: ✗.

## Data Availability

The data that support the findings of this study are available from the corresponding authors upon reasonable request.

## Supplementary Materials

The following are available online at https://www.mdpi.com/article/10.3390/jof7110985/s1, Supplementary File S1.
