# Supplementary material for: Global Prevalence of COVID-19-Associated Mucormycosis (CAM): Living Systematic Review and Meta-Analysis
Source: J Fungi (Basel). 2021 Nov 18;7(11):985. doi: 10.3390/jof7110985 (PMC8624337; doi:10.3390/jof7110985)
Supplement: Supplementary file 1 [file jof-07-00985-s001.zip › jof-1455606-supplementary-done.pdf]

Journal of Fungi

MDPI

Supplementary

Table S1. (PRISMA 2020 Checklist).

**TITLE**			
Title	1	Identify the report as a systematic review.	1
**ABSTRACT**			
Abstract	2	See the PRISMA 2020 for Abstracts checklist.	1
**INTRODUCTION**			
Rationale	3	Describe the rationale for the review in the context of existing knowledge.	2
Objectives	4	Provide an explicit statement of the objective(s) or question(s) the review addresses.	2
**METHODS**			
Eligibility criteria	5	Specify the inclusion and exclusion criteria for the review and how studies were grouped for the syntheses.	3
Information sources	6	Specify all databases, registers, websites, organisations, reference lists and other sources searched or consulted to identify studies. Specify the date when each source was last searched or consulted.	3
Search strategy	7	Present the full search strategies for all databases, registers and websites, including any filters and limits used.	Supplement
Selection process	8	Specify the methods used to decide whether a study met the inclusion criteria of the review, including how many reviewers screened each record and each report retrieved, whether they worked independently, and if applicable, details of automation tools used in the process.	3
Data collection process	9	Specify the methods used to collect data from reports, including how many reviewers collected data from each report, whether they worked independently, any processes for obtaining or confirming data from study investigators, and if applicable, details of automation tools used in the process.	3
Data items	10a	List and define all outcomes for which data were sought. Specify whether all results that were compatible with each outcome domain in each study were sought (e.g. for all measures, time points, analyses), and if not, the methods used to decide which results to collect.	3
	10b	List and define all other variables for which data were sought (e.g. participant and intervention characteristics, funding sources). Describe any assumptions made about any missing or unclear information.	3
Study risk of bias assessment	11	Specify the methods used to assess risk of bias in the included studies, including details of the tool(s) used, how many reviewers assessed each study and whether they worked independently, and if applicable, details of automation tools used in the process.	3
Effect measures	12	Specify for each outcome the effect measure(s) (e.g. risk ratio, mean difference) used in the synthesis or presentation of results.	4
Synthesis methods	13a	Describe the processes used to decide which studies were eligible for each synthesis (e.g. tabulating the study intervention characteristics and comparing against the planned groups for each synthesis (item #5)).	3
	13b	Describe any methods required to prepare the data for presentation or synthesis, such as handling of missing summary statistics, or data conversions.	4
	13c	Describe any methods used to tabulate or visually display results of individual studies and syntheses.	4
	13d	Describe any methods used to synthesize results and provide a rationale for the choice(s). If meta-analysis was performed, describe the model(s), method(s) to identify the presence and extent of statistical heterogeneity, and software package(s) used.	4
	13e	Describe any methods used to explore possible causes of heterogeneity among study results (e.g. subgroup analysis, meta-regression).	4
	13f	Describe any sensitivity analyses conducted to assess robustness of the synthesized results.	4
Reporting bias assessment	14	Describe any methods used to assess risk of bias due to missing results in a synthesis (arising from reporting biases).	4
Certainty assessment	15	Describe any methods used to assess certainty (or confidence) in the body of evidence for an outcome.	4
**RESULTS**			
Study selection	16a	Describe the results of the search and selection process, from the number of records identified in the search to the number of studies included in the review, ideally using a flow diagram.	4, Fig.1
	16b	Cite studies that might appear to meet the inclusion criteria, but which were excluded, and explain why they were excluded.	Supplement Table 3
Study characteristics	17	Cite each included study and present its characteristics.	5, Table 1
Risk of bias in studies	18	Present assessments of risk of bias for each included study.	5, Table 2
Results of individual studies	19	For all outcomes, present, for each study: (a) summary statistics for each group (where appropriate) and (b) an effect estimate and its precision (e.g. confidence/credible interval), ideally using structured tables or plots.	7-8, Fig.2-5
Results of syntheses	20a	For each synthesis, briefly summarise the characteristics and risk of bias among contributing studies.	7-8
	20b	Present results of all statistical syntheses conducted. If meta-analysis was done, present for each the summary estimate and its precision (e.g. confidence/credible interval) and measures of statistical heterogeneity. If comparing groups, describe the direction of the effect.	7-8
	20c	Present results of all investigations of possible causes of heterogeneity among study results.	7-8
	20d	Present results of all sensitivity analyses conducted to assess the robustness of the synthesized results.	7-8
Reporting biases	21	Present assessments of risk of bias due to missing results (arising from reporting biases) for each synthesis assessed.	NA
Certainty of evidence	22	Present assessments of certainty (or confidence) in the body of evidence for each outcome assessed.	8, Table 3
**DISCUSSION**			
Discussion	23a	Provide a general interpretation of the results in the context of other evidence.	9
	23b	Discuss any limitations of the evidence included in the review.	10

	23c	Discuss any limitations of the review processes used.	10
	23d	Discuss implications of the results for practice, policy, and future research.	10
**OTHER INFORMATION**			
Registration and protocol	24a	Provide registration information for the review, including register name and registration number, or state that the review was not registered.	NA
	24b	Indicate where the review protocol can be accessed, or state that a protocol was not prepared.	NA
	24c	Describe and explain any amendments to information provided at registration or in the protocol.	NA
Support	25	Describe sources of financial or non-financial support for the review, and the role of the funders or sponsors in the review.	10
Competing interests	26	Declare any competing interests of review authors.	10
Availability of data, code and other materials	27	Report which of the following are publicly available and where they can be found: template data collection forms; data extracted from included studies; data used for all analyses; analytic code; any other materials used in the review.	10

From: Page MJ, McKenzie JE, Bossuyt PM, Boutron I, Hoffmann TC, Mulrow CD, et al. The PRISMA 2020 statement: an updated guideline for reporting systematic reviews. BMJ 2021;372:n71. doi: 10.1136/bmj.n71.

Table S2. (MOOSE Checklist).

Item No	Recommendation	Reported on Page No
	Reporting of background should include	
1	Problem definition	1
2	Hypothesis statement	2
3	Description of study outcome(s)	2
4	Type of exposure or intervention used	2
5	Type of study designs used	2-3
6	Study population	2-3
	Reporting of search strategy should include	
7	Qualifications of searchers (eg, librarians and investigators)	2
8	Search strategy, including time period included in the synthesis and key words	2-3, supplement
9	Effort to include all available studies, including contact with authors	3
10	Databases and registries searched	3
11	Search software used, name and version, including special features used (eg, explosion)	3-4
12	Use of hand searching (eg, reference lists of obtained articles)	5
13	List of citations located and those excluded, including justification	5, supplement
14	Method of addressing articles published in languages other than English	NA
15	Method of handling abstracts and unpublished studies	5
16	Description of any contact with authors	Not Required
	Reporting of methods should include	
17	Description of relevance or appropriateness of studies assembled for assessing the hypothesis to be tested	5
18	Rationale for the selection and coding of data (eg, sound clinical principles or convenience)	5
19	Documentation of how data were classified and coded (eg, multiple raters, blinding and interrater reliability)	6
20	Assessment of confounding (eg, comparability of cases and controls in studies where appropriate)	6
21	Assessment of study quality, including blinding of quality assessors, stratification or regression on possible predictors of study results	6
22	Assessment of heterogeneity	6
23	Description of statistical methods (eg, complete description of fixed or random effects models, justification of whether the chosen models account for predictors of study results, dose-response models, or cumulative meta-analysis) in sufficient detail to be replicated	6-7
24	Provision of appropriate tables and graphics	6
	Reporting of results should include	
25	Graphic summarizing individual study estimates and overall estimate	7 Fig.2-5
26	Table giving descriptive information for each study included	7, Table 1
27	Results of sensitivity testing (eg, subgroup analysis)	8
28	Indication of statistical uncertainty of findings	8

	**Reporting of discussion should include**	
**29**	**Quantitative assessment of bias (eg, publication bias)**	NA
30	Justification for exclusion (eg, exclusion of non-English language citations)	NA
31	Assessment of quality of included studies	Table 2
	Reporting of conclusions should include	
32	Consideration of alternative explanations for observed results	8

33	Generalization of the conclusions (ie, appropriate for the data presented and within the domain of the literature review)	9-10
34	Guidelines for future research	10
35	Disclosure of funding source	10

From: Stroup DF, Berlin JA, Morton SC, et al, for the Meta-analysis Of Observational Studies in Epidemiology (MOOSE) Group. Meta-analysis of Observational Studes in Epidemiology. A Proposal for Reporting. *JAMA*. 2000;283(15):2008-2012. doi: 10.1001/jama.283.15.2008.

MEDLINE(R) ALL <1946 to August 19, 2021> (Ovid)
Search was conducted on 20th August 2021 at 2:05 pm (CET).

#	**search string**	**# of results**
**1**	exp Coronavirus/	90036
**2**	exp Coronavirus Infections/	110177
**3**	(coronavirus* or corona virus* or OC43 or NL63 or 229E or HKU1 or HCoV* or ncov* or covid* or sars-cov* or sarscov* or Sars-coronavirus* or Severe Acute Respiratory Syndrome Coronavirus* or "Kawasaki like paediatric inflammatory multisystem syndrome" or "Kawasaki like pediatric inflammatory multisystem syndrome" or "PIMS-TS" or "Kawa-COVID-19" or "MIS-C" or "multisystem inflammatory syndrome in children" or pediatric multisystem inflammatory disease).mp.	184833
**4**	(or/1-3) and ((20191* or 202*).dp. or 20190101:20301231.(ep).) (147001)	171857
**5**	4 not (SARS or SARS-CoV or MERS or MERS-CoV or Middle East respiratory syndrome or camel* or dromedar* or equine or coronary or coronal or covidence* or covidien or influenza virus or HIV or bovine or calves or TGEV or feline or porcine or BCoV or PED or PEDV or PDCoV or FIPV or FCoV or SADS-CoV or canine or CCov or zoonotic or avian influenza or H1N1 or H5N1 or H5N6 or IBV or murine corona*).mp.	62522
**6**	((pneumonia or covid* or coronavirus* or corona virus* or ncov* or 2019-ncov or sars*).mp. or exp pneumonia/) and Wuhan.mp.	5708
**7**	(2019-ncov or ncov19 or ncov-19 or 2019-novel CoV or sars-cov2 or sars-cov-2 or sarscov2 or sarscov-2 or SARS-2-nCoV or SARS-2-Cov or SARS-COV-19 or Sars-coronavirus2 or Sars-coronavirus-2 or SARS 2 coronavirus* or Severe Acute Respiratory Syndrome-CoV-2 or SARS-like coronavirus* or coronavirus-19 or covid19 or covid-19 or covid 2019 or ((novel or new or nouveau) adj2 (CoV or nCoV or covid or coronavirus* or corona virus or Pandemi*2)) or ((covid or covid19 or covid-19 or SARS-CoV-2) and pandemic*2) or (coronavirus* and pneumonia)).mp.	169435
**8**	(COVID-19 or SARS-CoV-2).rx,px,ox,rn. or (COVID-19 or COVID-19 serotherapy or ORF7b protein, SARS-CoV-2 or ORF6 protein, SARS-CoV-2 or ORF8 protein, SARS-CoV-2 or pediatric multisystem inflammatory disease, COVID-19 related or envelope protein, SARS-CoV-2 or ORF7a protein, SARS-CoV-2 or spike protein, SARS-CoV-2 or ORF3a protein, SARS-CoV-2 or COVID-19 drug treatment or severe acute respiratory syndrome coronavirus 2 or membrane protein, SARS-CoV-2 or ORF1ab polyprotein, SARS-CoV-2 or nucleocapsid protein, Coronavirus or COVID-19 vaccine or COVID-19 diagnostic testing).os,ps,rn,rs.	10892
**9**	("32185863" or "32172715" or "32227595" or "32140676" or "32246156" or "32267941" or "32176889" or "32169616" or "32265186" or "32253187" or "32152148" or "32053580" or "32179788" or "32213260" or "32205350" or "32188729" or "32152361" or "32277065" or "32088947" or "32240583" or "31917786" or "32127714" or "32047315" or "32020111" or "32240632" or "32243118" or "32267344" or "32239781" or "32396977" or "32402130" or "32243299" or "32807526" or "32344395" or "32403202" or "32389714" or "32416016" or "32405099" or "32976849" or "32685966" or "33221888" or "32379271" or "32188728" or "32221976" or "32417321" or "32489438" or "32332959" or "32943452" or "32807525" or "32826274" or "32898560" or "32293023" or "33159926" or "32919952" or "32835716" or "32619499" or "32663524" or "32392627" or "32392625" or "33037657" or "32777045" or "32521569" or "32492200" or "32930765" or "33075143" or "32237249" or "32683439" or "32495994" or "32344447" or "32896006" or "32240549" or "32438448" or "32425477" or "32951095" or "32274794" or "32750178" or "32463935" or "32428286" or "32491981" or "32930748" or "32119409" or "32432657" or "33003176" or "32459319" or "32822920" or "32878290" or "32270498" or "32250493" or "32512243" or "32837399" or "32426074" or "32199942" or "32839969" or "32639522" or "33073717" or "32502134" or "32334003" or "32510470" or "32819741" or "32309248" or "32243951" or "32378772" or "32835361" or "32962779" or "32916324" or "32785973" or "32272221" or "32299207" or "33044515" or "33134955" or "32970917" or "32407438" or "32513790" or "32439468" or "33063036" or "33077677" or "32406056" or "32716821" or "32588590" or "32239757" or "32829902" or "32807521" or "32379350" or "33125767" or "32829731" or "32988821" or "32780977" or "32648633" or "32829907" or "32330635" or "32692998" or "33013067" or "33010706" or "32502292" or "32780969" or "32998780" or "32754731" or "32639607" or "32233030" or "32953429" or "32246897" or "32955802" or "32425490" or "32418270" or "32445255" or "32775945" or "32775948" or "32775953" or "32407043").ui.	151
**10**	or/5-9	172345
**11**	10 and 20191201:20301231.(dt).	170194
**12**	exp Zygomycosis/	4538
**13**	mucormycos#s.mp.	5206
**14**	Mucormycose.mp.	98

**15**	mucoromycos#s.mp.	6
**16**	zygomycos#s.mp.	1426
**17**	(black fungus or black fungi).mp.	210
**18**	exp Mucorales/	6677
**19**	Mucorales.mp.	2036
**20**	mucoralean.mp.	73
**21**	Absidia.mp.	566
**22**	Cunninghamella.mp.	773
**23**	Mortierella.mp.	770
**24**	Mucor.mp.	3411
**25**	Apophysomyces.mp.	148
**26**	Saksenaea.mp.	104
**27**	Rhizopus.mp.	4248
**28**	Rhizomucor.mp.	700
**29**	Lichtheimia.mp.	195
**30**	Cokeromyces.mp.	24
**31**	Actinomucor.mp.	59
**32**	Syncephalastrum.mp.	163
**33**	12 or 13 or 14 or 15 or 16 or 17 or 18 or 19 or 20 or 21 or 22 or 23 or 24 or 25 or 26 or 27 or 28 or 29 or 30 or 31 or 32	14775
**34**	11 and 33	164

Embase <1974 to 2021 August 19> (Ovid)
Search was conducted on 20th August 2021 at 2:00 pm (CET).

#	**search string**	**# of results**
**1**	exp Coronavirus/	61250
**2**	exp Coronavirus Infections/	159534
**3**	(coronavirus* or corona virus* or OC43 or NL63 or 229E or HKU1 or HCoV* or ncov* or covid* or sars-cov* or sarscov* or Sars-coronavirus* or Severe Acute Respiratory Syndrome Coronavirus* or "Kawasaki like paediatric inflammatory multisystem syndrome" or "Kawasaki like pediatric inflammatory multisystem syndrome" or "PIMS-TS" or "Kawa-COVID-19" or "MIS-C" or "multisystem inflammatory syndrome in children" or pediatric multisystem inflammatory disease).mp.	202998
**4**	(or/1-3) and 20190101:20301231.(dc).	182384
**5**	4 not (SARS or SARS-CoV or MERS or MERS-CoV or Middle East respiratory syndrome or camel* or dromedar* or equine or coronary or coronal or covidence* or covidien or influenza virus or HIV or bovine or calves or TGEV or feline or porcine or BCoV or PED or PEDV or PDCoV or FIPV or FCoV or SADS-CoV or canine or CCov or zoonotic or avian influenza or H1N1 or H5N1 or H5N6 or IBV or murine corona*).mp.	111905
**6**	((pneumonia or covid* or coronavirus* or corona virus* or ncov* or 2019-ncov or sars*).mp. or exp pneumonia/) and Wuhan.mp.	5948
**7**	(coronavirus disease 2019 or 2019-ncov or 2019nCoV or ncov2019 or ncov19 or ncov-19 or HCoV-19 or 2019-novel CoV or severe acute respiratory syndrome coronavirus 2 or sars2 or sars 2 or sars-cov2 or sars-cov-2 or sarscov2 or sarscov-2 or Sars-coronavirus2 or Sars-coronavirus-2 or SARS-CoV-19 or SARS-2-nCoV or SARS-2-Cov or SARS 2 coronavirus* or Severe Acute Respiratory Syndrome-CoV-2 or SARS-like coronavirus* or coronavirus-19 or covid19 or covid-19 or covid 2019 or ((novel or new or nouveau) adj2 (CoV or nCoV or covid or coronavirus* or corona virus or Pandemi*2)) or ((covid or covid19 or covid-19 or SARS-CoV-2) and pandemic*2) or (coronavirus* and pneumonia)).mp.	178931
**8**	(coronavirus disease 2019 or severe acute respiratory syndrome coronavirus 2).sh,dj.	145673
**9**	("630575119" or "630830186" or "630941329" or "631043694" or "631260659" or "631272428" or "631272880" or "631286076" or "631290163" or "631308782" or "631324397" or "631352500" or "631416440" or "631431802" or "631452886" or "631456079" or "631457551" or "631462438" or "631462876" or "631465538" or "631465685" or "631469310" or "2004499662" or "2004505338" or "2005280837" or "2005387675" or "2005408544" or "2005484987" or "2005549151").an.	16
**10**	(or/6-9) and 20191201:20301231.(dc).	175825
**11**	5 or 10	178067
**12**	zygomycosis/ or mucormycosis/	7603
**13**	mucormycos#s.mp.	7598
**14**	Mucormycose.mp.	113
**15**	mucoromycos#s.mp.	7
**16**	zygomycos#s.mp.	1696
**17**	(black fungus or black fungi).mp.	225

**18**	exp Mucorales/	11429
**19**	Mucorales.mp.	1898
**20**	mucoralean.mp.	91
**21**	Absidia.mp.	862
**22**	Cunninghamella.mp.	1138
**23**	Mortierella.mp.	905
**24**	Mucor.mp.	5061
**25**	Apophysomyces.mp.	208
**26**	Saksenaea.mp.	139
**27**	Rhizopus.mp.	6457
**28**	Rhizomucor.mp.	1224
**29**	Lichtheimia.mp.	446
**30**	Cokeromyces.mp.	29
**31**	Actinomucor.mp.	73
**32**	Syncephalastrum.mp.	292
**33**	12 or 13 or 14 or 15 or 16 or 17 or 18 or 19 or 20 or 21 or 22 or 23 or 24 or 25 or 26 or 27 or 28 or 29 or 30 or 31 or 32	20174
**34**	11 and 33	159

Cochrane COVID-19 Study Register
Search was conducted on 20th August 2021 at 2:10 pm (CET).

#	**search string**	**# of results**
**1**	mucormycosis OR mucormycoses OR mucormycose OR mucoromycosis OR mucoromycoses OR zygomycosis OR zygomycoses OR "black fungus" OR "black fungi" OR Mucorales OR mucoralean OR Absidia OR Cunninghamella OR Mortierella OR Mucor OR Apophysomyces OR Saksenaea OR Rhizopus OR Rhizomucor OR Lichtheimia OR Cokeromyces OR Actinomucor OR Syncephalastrum	74

WHO COVID-19 database
Search was conducted on 20th August 2021 at 2:20 pm (CET).

#	**search string**	**# of results**
**1**	tw:(MH:C01.150.703.980* OR MH: B01.300.300.500* OR mucormycosis OR mucormycoses OR mucormycose OR mucoromycosis OR mucoromycoses OR zygomycosis OR zygomycoses OR "black fungus" OR "black fungi" OR Mucorales OR mucoralean OR Absidia OR Cunninghamella OR Mortierella OR Mucor OR Apophysomyces OR Saksenaea OR Rhizopus OR Rhizomucor OR Lichtheimia OR Cokeromyces OR Actinomucor OR Syncephalastrum)	196

Table S3. (List of excluded articles).

**Author, year**	**Title**	**Reason for exclusion**
Frías-De-León et al. 2021 [1]	Epidemiology of Systemic Mycoses in the COVID-19 Pandemic	Study design
Desai et al. 2021 [2]	Epidemiology, Clinical Features and Management of Rhino Orbital Mucormycosis in Post COVID 19 Patients	Outcome not of interest
Danion et al. 2021 [3]	High mortality of COVID-19 associated mucormycosis in France: a nationwide retrospective study	Outcome not of interest
Ismaiel et al. 2021 [4]	The impact of COVID-19 outbreak on the incidence of acute invasive fungal rhinosinusitis	Outcome not of interest
Siluvai et al. 2021 [5]	Incidence of Mucormycosis in a Tertiary Care Hospital During Covid First Wave-A Retrospective Study	Outcome not of interest
Banerjee et al. 2021 [6]	Mucormycosis and COVID-19 an epidemic in a pandemic?	Study design
Fekkar et al. 2021 [7]	Occurrence of invasive pulmonary fungal infections in patients with severe COVID-19 admitted to the ICU	Outcome not of interest
Moorthy et al. 2021 [8]	SARS-CoV-2, Uncontrolled Diabetes and Corticosteroids-An Unholy Trinity in Invasive	Outcome not of interest

	Fungal Infections of the Maxillofacial Region? A Retrospective, Multi-centric Analysis	
Fouad et al. 2021 [9]	Spike in Rhino-Orbital-Cerebral Mucormycosis Cases Presenting to a Tertiary Care Center During the COVID-19 Pandemic	Outcome not of interest

References

1. Frías-De-León MG, Pinto-Almazán R, Hernández-Castro R, García-Salazar E, Meza-Meneses P, Rodríguez-Cerdeira C, Arenas R, Conde-Cuevas E, Acosta-Altamirano G, Martínez-Herrera E. Epidemiology of systemic mycoses in the COVID-19 pandemic. *J. Fungi*. **2021**;*7*, 556.
2. Desai EJ, Pandya A, Upadhya I, Patel T, Banerjee S, Jain V. Epidemiology, Clinical Features and Management of Rhino Orbital Mucormycosis in Post COVID 19 Patients. *Indian J Otolaryngol*. **2021**, 1–5.
3. Danion F, Letscher-Bru V, Guitard J, Sitbon K, Delliere S, Angoulvant A, Desoubeaux G, Botterel F, Bellanger AP, Gargala G, Uhel F. High mortality of COVID-19 associated mucormycosis in France: a nationwide retrospective study. medRxiv, accessed on 1 January 2021.
4. Ismaiel WF, Abdelazim MH, Eldsoky I, Ibrahim AA, Alsobky ME, Zafan E, Hasan A. The impact of COVID-19 outbreak on the incidence of acute invasive fungal rhinosinusitis. *Am. J. Otolaryngol*., **2021**;*42*, 103080.
5. Siluvai Arulappan LA. Incidence of Mucormycosis in a Tertiary Care Hospital During Covid First Wave-A Retrospective Study. *Indian J Otolaryngol*. **2021**, 1–6.
6. Banerjee I, Robinson J, Asim M, Sathian B, Banerjee I. Mucormycosis and COVID-19 an epidemic in a pandemic?. *Nepal J. Epidemiology*. **2021**;*11*, 1034.
7. Fekkar A, Lampros A, Mayaux J, Poignon C, Demeret S, Constantin JM, Marcelin AG, Monsel A, Luyt CE, Blaize M. Occurrence of invasive pulmonary fungal infections in patients with severe COVID-19 admitted to the ICU. *Am. J. Respir. Crit. Care Med*. **2021**, *203*, 307–17.
8. Moorthy A, Gaikwad R, Krishna S, Hegde R, Tripathi KK, Kale PG, Rao PS, Haldipur D, Bonanthaya K. SARS-CoV-2, Uncontrolled Diabetes and Corticosteroids—An Unholy Trinity in Invasive Fungal Infections of the Maxillofacial Region? A Retrospective, Multi-centric Analysis. *J. Oral Maxillofac. Surg*. **2021**, 1–8.
9. Fouad YA, Abdelaziz TT, Askoura A, Saleh MI, Mahmoud MS, Ashour DM, Ashour MM. Spike in rhino-orbital-cerebral mucormycosis cases presenting to a tertiary care center during the COVID-19 pandemic. *Front. Med*., **2021**, *8*.
